# Postoperative infection caused by *Acinetobacter baumannii* misdiagnosed as a free-living amoeba species in a humeral head hemiarthroplasty patient: a case report

**DOI:** 10.1186/s40249-018-0408-5

**Published:** 2018-03-31

**Authors:** Jiaxin Tang, Huaimin Zhu, Li Cai, Tingting Tang, Jian Tang, Yuehua Sun, Ming Liu, Kerong Dai, Zhiguang Qiao, Chao Yu

**Affiliations:** 1 Shanghai Key Laboratory of Orthopaedic Implants, Department of Orthopaedic Surgery, Shanghai Ninth People’s Hospital, Shanghai Jiao Tong University School of Medicine, Shanghai, 200011 China; 2 Department of Microbiology and Parasitology, Second Military Medical University, Shanghai, 200433 China; 3 Shanghai Municipal Center for Disease Control & Prevention, Shanghai, 200336 China; 4 Medical 3D Printing Innovation Research Center, Shanghai Jiao Tong University School of Medicine, Shanghai, 200125 China; 5 Engineering Research Center of Digital Medicine and Clinical Translation, Ministry of Education, Shanghai, 200030 China

**Keywords:** *Acinetobacter baumannii*, Free-living amoebae, Hemiarthroplasty, Case report

## Abstract

**Background:**

*Acinetobacter baumannii* is ubiquitous, facultative intracellular, and opportunistic bacterial pathogen. Its unique abilities allow it to survive in a diverse range of environments, including health care settings, leading to nosocomial infections. And its exceptional ability to develop resistance to multiple antibiotics leaves few drug options for treatment. It has been recognized as a leading cause of nosocomial pneumonia and bacteremia over the world.

**Case presentation:**

In this case, a 73-year-old woman presented with a Neer Group VI proximal humeral fracture. Six hours after a successfully performed hemiarthroplasty, she developed continuous fever. Clinical examination revealed that the vitals were regular. Laboratory and radiographic examinations revealed only elevated procalcitonin levels. Blood culture revealed no bacterial or fungal growth. Cooling treatment and empirical broad-spectrum antibiotic therapy showed no apparent effect.

**Conclusions:**

We report a postoperative infection caused by *Acinetobacter baumannii*. The infectious pathogen was identified via molecular DNA sequencing and was initially misidentified as a free-living amoeba species upon microscopic examinations. The patient was mistreated with antiamebic combination therapy. Her symptoms persisted for over 4 months and were eventually followed by her death.

**Electronic supplementary material:**

The online version of this article (10.1186/s40249-018-0408-5) contains supplementary material, which is available to authorized users.

## Multilingual abstracts

Please see Additional file 1 for translations of the abstract into the five official working laguages of the United Nations.

## Background

During the past few decades, *Acinetobacter baumannii* has received significant attention from scientific and medical communities [1–5]. Its unique abilities, such as intrinsically resistance to desiccation, allow it to survive in a diverse range of environments, including health care settings, leading to nosocomial infections and troublesome outbreaks [6]. As a nosocomial pathogen which is most commonly involved in hospital infections, *A. baumannii* has an exceptional ability to develop resistance to multiple antibiotics, leaving few drug options for treatment [3, 6–8]. Inappropriate initial antimicrobials were strongly associated with increased mortality for *A. baumannii* infections [9]. In various parts over the globe, it has been recognized as a leading cause of nosocomial pneumonia and bacteremia [10–13].

Clinically, *A. baumannii* usually affects fragile, immunocompromised patients, especially those who suffer from burns, have trauma, or are in the intensive care units (ICUs) [14]. Those infected are commonly associated with mechanical ventilation, intravenous and urinary catheterization, surgery, invasive procedures, and prolonged broad-spectrum antimicrobials [15–20]. Clinical reports suggest that *Acinetobacter* can cause serious, life-threatening infections [6].

In this case report, we describe a rare case of infection caused by *Acinetobacter baumannii* misdiagnosed as a free-living amoeba (FLA) species and mistreated with an antiamebic combination therapy with metronidazole, fluconazole, compounded sulfamethoxazole and sulfadiazine (COSMZ), dihydroartemisinin and piperaquine phosphate combined (Duo-Cotecxin), and meropenem in a 73-year-old woman admitted to the Department of Orthopaedic Surgery, Shanghai Ninth People’s Hospital, Shanghai Jiao Tong University School of Medicine, Shanghai, China.

## Case presentation

**Fig. 1:**
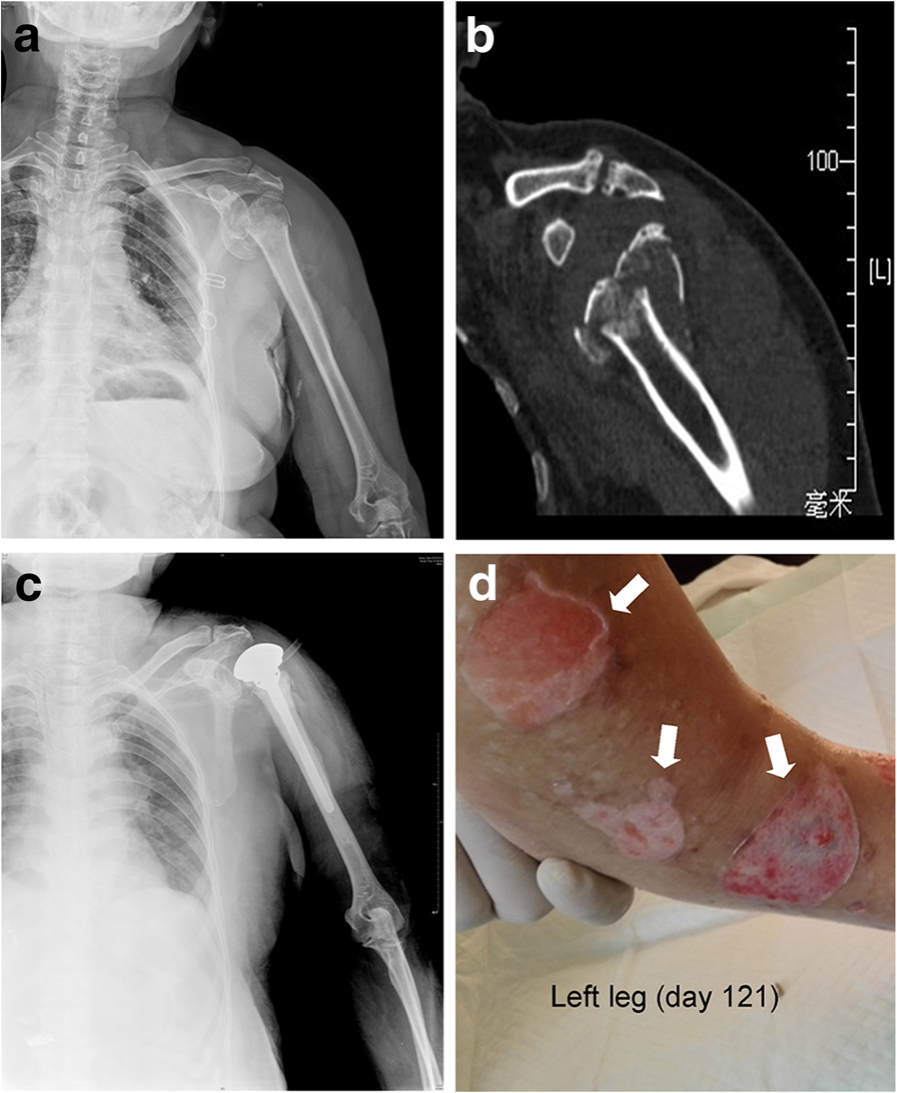
Radiograpic findings of the left shoulder and ruptrured blisters in the left leg. **a:** Plain radiograph showing the patient’s left shoulder at admission. **b:** CT scans of the same area. **c:** Plain radiograph of the left shoulder after hemiarthroplasty surgery. **d:** During the treatment failure stage, the patient developed multiple blisters in the left leg. Defects in the skin developed after the blisters ruptured (arrows)

A 73-year-old woman presented with severe pain in the left upper extremity following a ground-level fall at her home. Physical examination revealed shoulder deformity and restricted range of motion, crepitus from the fracture fragments and extensive ecchymosis to the elbow on her left shoulder. No neurovascular impairment was found. Plain radiographs and computed tomography (CT) scans showed a Neer Group VI proximal humeral fracture (Fig. 1a-b) [21]. Her medical history was significant for lacunar infarction, hypertension, type 2 diabetes and schizophrenia, which were all controlled with oral medications. She was diagnosed with schizophrenia by the Shanghai Mental Health Center. Medical records showed that the patient had irregular fever along with symptoms of schizophrenia such as impulsive tendencies of self-mutilation and aggressive behaviors over decades. Additionally, the patient had a history of eating raw clams (*Tegillarca granosa*) and years of working barefoot in paddy fields without wearing protective clothing.

In this case, the patient stayed in hospital for 134 days. On the basis of the maximum body temperature and laboratory examinations, hospitalization was divided into three stages, namely, fever of unknown origin (FUO) stage (days 1–36), antiamebic treatment (AT) stage (days 37–85), and treatment failure (TF) stage (days 86–134) (Additional files 2 and 3).

### Fever of unknown origin stage (FUO, days 1–36)

**Fig. 2:**
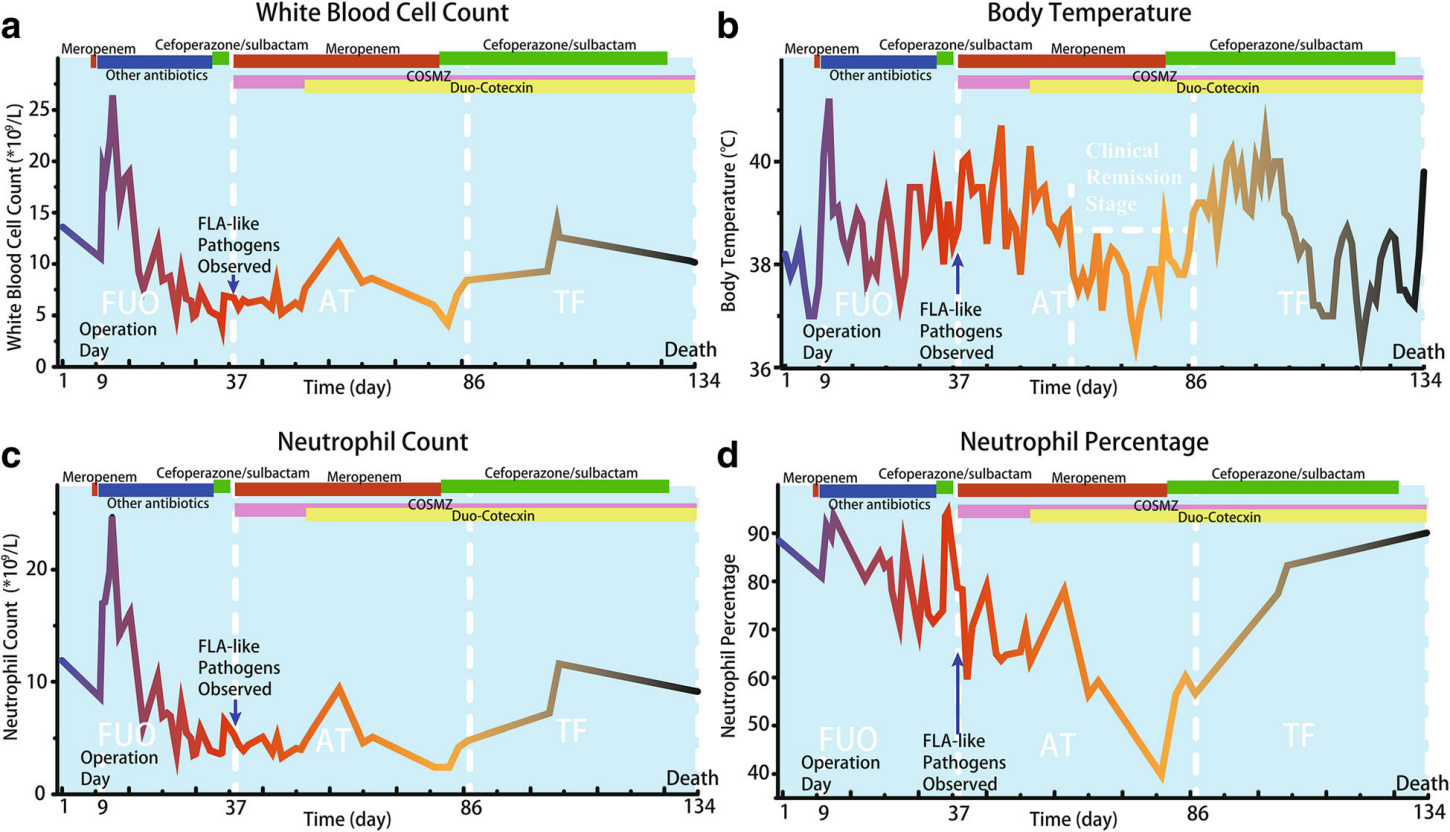
Line chart demonstrate **a**: Blood white blood cell count, **b:** Maximum body temperature, **c:** neutrophil count, and **d:** Neutrophil percentage during hospitalization. The time periods when antibiotics and antiamebics were used are shown

After surgery contraindications were ruled out, the patient underwent a hemiarthroplasty surgery performed by an experienced orthopedic surgeon on day 9 (Fig. 1c). The operation was successfully performed without any intraoperative complications. Six hours after the surgery, she developed continuous fever (body temperature 40.3 °C) (Fig. 2b). Measures for lowering body temperature were taken immediately. Physical examination showed her vitals were normal. The patient had negative Kernig’s sign and neck stiffness. Motor, sensory and cranial nerve examinations were within normal limits. Laboratory and radiographic examinations revealed no obvious evidence of infection, except for elevated procalcitonin levels (0.38 ng/ml, normal levels 0–0.1 ng/ml). Blood (1,3)-*β*-D-glucan assays were taken 5 times during this stage, and the results were negative. Blood culture was performed several times and revealed no bacterial or fungal growth. Meropenem, cefoperazone/sulbactam, cefathiamidine, vancomycin, azithromycin, fluconazole, ceftazidime, phosphonomycin and linezolid were used as mono- or combination antibiotic therapy successively. The operated upper limb healed uneventfully. However, cooling treatment and empirical broad-spectrum antibiotic therapy showed no apparent effect.

### Antiamebic treatment stage (AT, days 37–85)

**Fig. 3:**
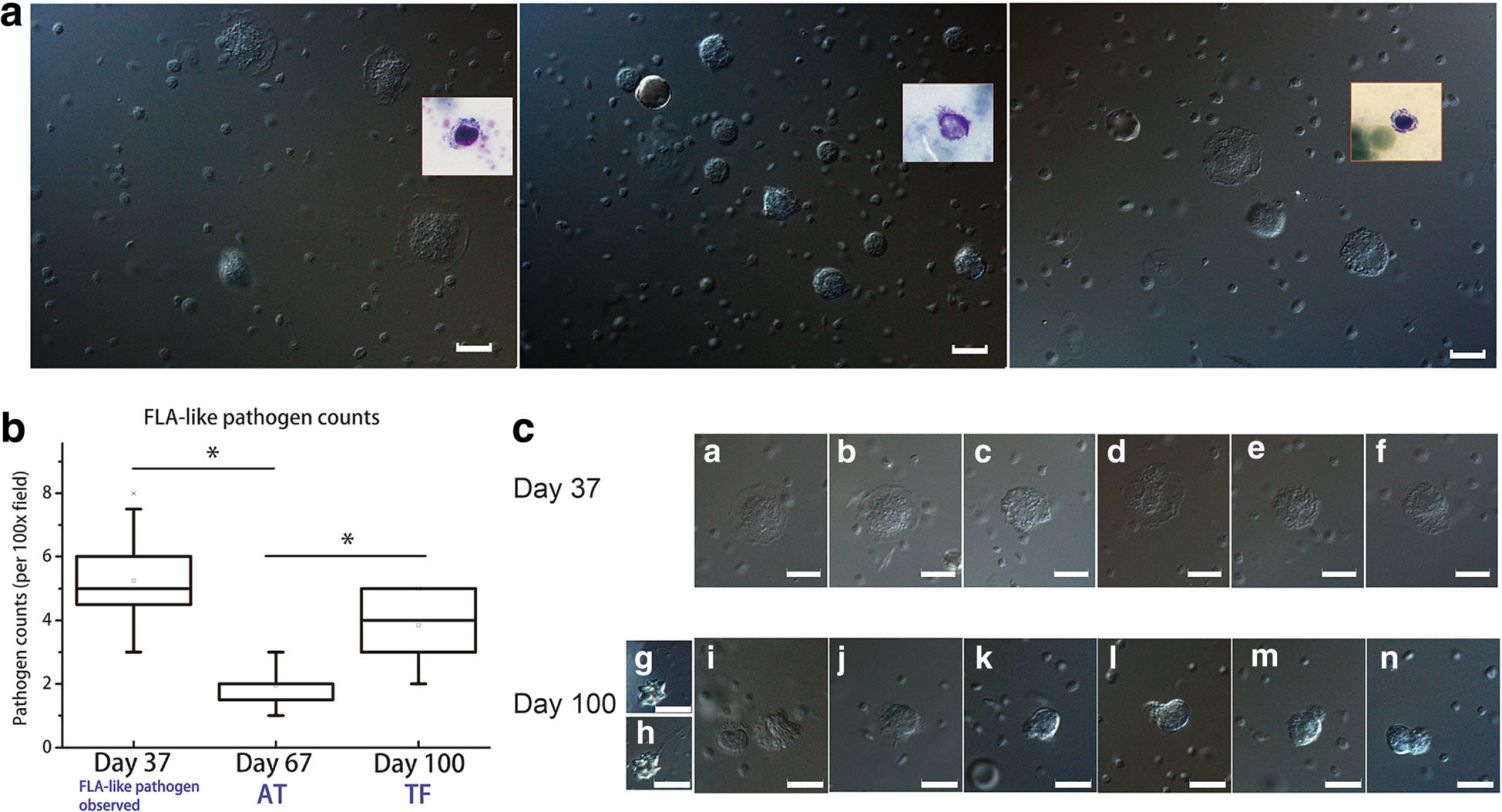
Microscopic findings in blood samples before and after treatment. **a**: Differential interference contrast images of the patient’s blood. The cells were discoid in shape, and the cytoplasmic humps were surrounded with flattened hyaline margins, some of which were fan-shaped. Insets are Giemsa-stained blood film images. **b**: Box chart showing FLA-like pathogen counts per high power magnification (100 ×) field in blood samples at three different time points. At each time point, 20 blood samples were sent for FLA pathogen counting. **c**: Light microscopic images of the patient’s blood before (*a*–*f*) and after treatment (*g*–*n*). After treatment, most forms are oval. *g–n:* Locomotive cells display short filopodia or conical non-branching pseudopodia arising from apical and/or lateral parts of the cell. *g, h:* Small cells showing refractive outline with single long (over 10 μm) filopodia. *i, j:* Large irregular cells with short (3–5 μm) filopodia. Scale bars: 10 μm

**Fig. 4:**
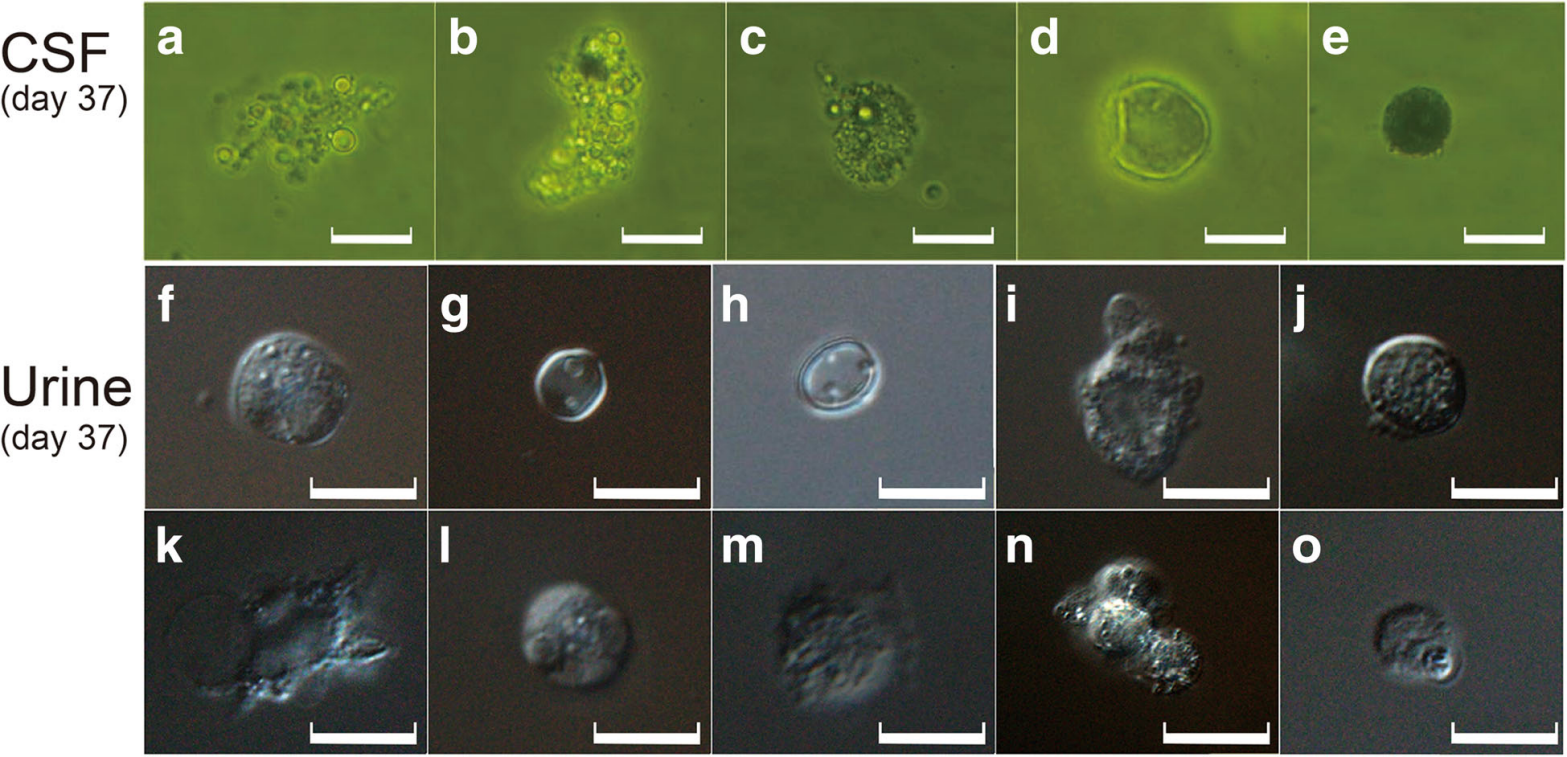
Microscopic findings in CSF and urine samples before treatment. **a:** Inverted microscopic images of the patient’s CSF. **a–c:** Irregularly shaped cells with vacuoles. **c:** Irregularly shaped cells with finger pseudopodia. **d:** Cells with transparent circular shells; no structures were observed. **e:** Black sphere. **b:** Differential interference contrast light microscopic images of the patient’s urine. **f–h, j, l, n:** Spherical cells. **k:** Cells with huge vacuoles. **m:** Cell with fan-shaped pseudopodia. Some “cells” cluster together. Scale bars: 10 μm

To investigate the etiology of continuous fever, blood, urine and cerebrospinal fluid (CSF) samples were sent for parasite detection. Surprisingly, large numbers of FLA-like pathogens were discovered microscopically (Fig. 3a, c; Fig. 4). The patient was initially diagnosed as having FLA infection and treated with antiamebic therapy.

On day 37, after the diagnosis of FLA infection was made, the patient was immediately started on a combination therapy with metronidazole, fluconazole, compounded sulfamethoxazole and sulfadiazine (COSMZ), and meropenem [22–26]. Meropenem was used in case that the patient was infected with other pathogens in addition to FLA. On day 52, Duo-Cotecxin (Beijing Holley-Cotec Pharmaceuticals Co., Ltd., China) was added to the antiamebic therapy. Additionally, the medical team attempted to obtain miltefosine, which did not arrive in time. From day 61–85, remission of clinical manifestations of the patient was observed. During this period, her mental status remained stable, no schizophrenia related symptoms appeared, and the body temperature was lower than 38.0 °C most of the time (Fig. 2b). Laboratory results were normal. A decrease in FLA-like pathogen from 5.25 to 1.95 counts under high power field at magnification (× 100) field in blood samples was also observed by microscopy (Fig. 3b).

### Treatment failure stage (TF, days 86–134)

Although a remission of clinical manifestations of the patient was observed for approximately 25 days (days 61 to 85), the patient’s condition deteriorated from day 86. Her body temperature was higher than 38.0 °C most of the time (Fig. 2b), and the FLA-like pathogen count in the blood samples as determined by microscopy increased again to 3.85 (Fig. 3b). Although frequently received blood transfusions (10 times comprised of 1 international unit of red cell suspension liquid and 100 ml of blood plasma, or 2 international unit of red cell suspension liquid and 200 ml of blood plasma each time), the patient became severely anemic. Her hospital course was complicated by refractory hypernatremia and hyperchloremia (sodium–153 mmol/L, chloride–114 mmol/L), leading to multiple blisters on the left leg (Fig. 1d). FLA-like pathogens were also detected in the blister fluid samples. She finally died of multiple organ failure and intractable fever on the 134th day of hospitalization.

### Pathogen morphology

Prior to observing in the microscope, blood, CSF and blood samples were processed with the physiological saline method of direct smears.

In the blood samples, many cells looked like “straw hats” (Fig. 3a). The cells were discoid in shape; the cytoplasmic humps were surrounded with flattened hyaline margins, some of which were fan-shaped. The surfaces of the cells were warty. The dimensions of these cells ranged from 10.2–25.4 × 11.8–26.5 μm with average dimensions of 17.8 × 19.3 μm. Smaller cells were easily observed. Owing to the granular mass on the surface, the nucleus could be not clearly seen. Locomotion was not visible in most microscopic fields. But sometimes cells displayed short blunt or fila-pseudopodia and slow deformation (Additional file 4).


Additional file 4: Video. Short video clip of FLA-like pathogen in blood sample under microscopic vision. Locomotion was not visible in most microscopic fields. But sometimes cells displayed short blunt or fila-pseudopodia and slow deformation. (MP4 18 599 kb)


In CSF samples, we found different cells that appeared irregular and had vacuoles (Fig. 4a-c) and finger pseudopodia (Fig. 4c). Some had circular shells that looked transparent, but no structures could be observed (Fig. 4d). Occasionally, black spheres were observed (Fig. 4e).

In urine samples, single cells displayed different morphological forms; some were spherical (Fig. 4f-h, j, l, n), whereas others had huge vacuoles (Fig. 4k) and/or possessed fan-shaped pseudopodia (Fig. 4m). Some “cells” clustered together.

After the antiamebic treatment, when the body temperature of the patient came down, the discoid-like unicellular body could not be detected in blood samples (Fig. 3c *g-n*). Most forms were oval; the cell bodies were approximately 13.0–16.1 μm long and 8.8–11.7 μm wide with average dimensions of 15.0 × 10.1 μm. Locomotive cells displayed short filopodia or conical non-branching pseudopodia arising from apical and/or lateral parts of the cell (Fig. 3c *k-n*). Furthermore, some small cells showing small, refractive outlines with single long (over 10 μm) filopodia (Fig. 3c *g, h*) and large irregularly shaped cells (more than 10 μm) with short (3–5 μm) filopodia (Fig. 3c *i, j*) could be seen occasionally.

The morphological characteristics of the pathogen resemble species of FLA, *Vannella* sp. [27], and another type of scale-bearing amoeba: *Cochliopodium* sp. [28], and with no similarity with known pathogenic amoeba such as *Acanthamoeba* sp.*, Balamuthia mandrillaris, Entamoeba histolytica, Naegleria fowleri, Sappinia diploidea*, etc. [29].

### Molecular identification

To confirm the morphological findings and further discover the cause of the infection, uncultured blood serum samples were sent for molecular identification. The blood serum samples were taken on day 44 and preserved in liquid nitrogen. Total genomic DNA was extracted from the samples and polymerase chain reaction (PCR) was performed using prokaryotic universal primers and eukaryotic universal primers (Additional file 5). Primer sequences were 16 s-27F 5′-AGAGTTTGATCATGGCTCAG-3′, 16 s-1390R 5′-ACGGGCGGTGTCTACAA-3′, 18S-F 5′-ACCTGGTTGATCCTGCCAGT-3′, 18S-R 5′-CTTGTTACGACTTTTACTTCC-3′, 18S-1080F 5′-GGGRAACTTACCAGGTCC-3′ and 18S-1578R 5′-GTGATRWGRTTTACTTRT-3′. The obtained sequencing data (1300 bp) using prokaryotic primers were compared with all published sequences in GenBank using BLASTn at National Center for Biotechnology Information (http://blast.ncbi.nlm.nih.gov/) and submitted to the GenBank database (Accession No. MG581460). Results showed that the sequence had a maximum of 80% identities with multiple *Acinetobacter baumannii* strains (Additional file 6). Comparison of the obtained sequence with *Acinetobacter baumannii* strain KAB05, complete genome (Accession No. CP017650.1) was shown in Additional file 7. Another sequencing data (1800 bp) were obtained using eukaryotic universal primers pair 18S-F/18S-R and had a maximum of 99% identities with Human Genomic sequences. No sequencing data were obtained using universal primers pair 18S-1080F/18S-1578R.

## Discussion

We report a case of postoperative infection in a humeral head hemiarthroplasty patient caused by *Acinetobacter baumannii*, which was identified via molecular DNA sequencing. The pathogen was initially misidentified as a FLA species.

*A. baumannii* is recognized as an opportunist pathogen which causes infections in fragile patients [30, 31]. Infections caused by *A. baumannii* are usually associated with defects in anatomical host defenses and alteration of normal host flora by exposure to broad-spectrum antibiotics [32], affecting mainly severely ill patients in the ICUs, and patients who have trauma or suffer from burns [3, 32]. The most common mode of transmission is via the hands of health care workers [33, 34]. The most common clinical manifestations of *A. baumannii* are nosocomial pneumonia and bacteremia [16, 35–40].

The exact time point of infection could not be established by now, but was speculated to be after the surgery. The route of infection in our patient is not clear. After scrutinizing the whole hospital course, we summarize the reasons for misdiagnosis and mistreatment into three points. First, clinical manifestations of this patient after infection were atypical. Common clinical manifestations of *A. baumannii* include nosocomial pneumonia, bacteremia, wound infections and osteomyelitis, urinary tract infections, endocarditis, and meningitis [16, 35–40]. The patient only developed continuous fever. The operated upper limb healed uneventfully, and none of the common manifestations of *A. baumannii* above were developed. Second, blood culture was performed several times and revealed no bacterial growth. Two antibiotic agents used in FUO stage, namely, meropenem and cefoperazone/sulbactam, had antimicrobial activity against *A. baumannii* [41] and were among current treatment options to *A. baumannii* infections [32, 42, 43]. Negative results of blood culture may due to the use of meropenem and cefoperazone/sulbactam, along with other antimicrobial treatments. The last and most important, the pathogens observed microscopically were FLA-like, rather than *A. baumannii*. Its microscopic characteristics resemble *Vannella* sp. [27], and another type of scale-bearing amoeba: *Cochliopodium* sp. [28]. Diagnosis of FLA infection was mainly based on the microscopic findings. In addition, after the initiation of antiamebic therapy, a clinical remission was observed, making us further believe that the causative pathogen was a FLA species.

The sequences obtained using prokaryotic universal primers were compared with all published sequences in GenBank. According to the results from molecular DNA sequencing, the final diagnosis is *A. baumannii* infection. However, this alone cannot explain the discovery of many FLA-like pathogens microscopically.

Two hypotheses were established for this, though neither can be verified by now. One hypothesis is that the patient was infected with two pathogens, FLA and *A. baumannii*. The FLA could be a *Vannella* species based on its morphology and may serve as host for *A. baumannii.* Some of the *Vannella* species are known to harbor bacterial pathogens in previous studies [44, 45]*.* The empirical broad-spectrum antibiotic therapy destroyed most of the FLA later, leaving *A. baumannii* in the predominant position. Another hypothesis is the strange FLA-like pathogens were actually the blood cells or parts of them, which were invaded by *A. baumannii* and serve as hosts for them. Bacteria living within eukaryotic cells are called intracellular bacteria. Obligate intracellular bacteria enter into cells and use host cell resources for their replication [46], while facultative intracellular bacteria can multiply both inside and outside host cells [47]. Intracellular bacteria generally reside directly in the host cytoplasm or in host-derived vacuoles [48], some can even invade eukaryotic nucleus [49]. They frequently hijack the host endocytic and secretory pathway, and structure the host a specialized cell for their replication [50]. Many intracellular bacteria are well-known pathogens, including *Legionella pneumophila* [51], *Francissella tularensis* [52], and *Mycobacterium tuberculosis* [53]. *A. baumannii* is also facultative intracellular bacteria [54], and had been reported to adhere and invade into human pulmonary cells, causing respiratory infections and pneumonia [55–57].

*A. baumannii* infections are commonly difficult to treat as the causative strains often have broad antimicrobial resistance. These *A. baumannii* strains are classified as multidrug-resistant (MDR), extensively drug-resistant (XDR) and pandrug-resistant (PDR) based on their antimicrobial susceptibility profile. MDR strains are resistant to three or more, XDR strains are resistant to all but one or two, and PDR are resistant to all classes of potentially effective antimicrobial agents [58].

Current treatment options to *A. baumannii* infections include carbapenems, sulbactam, tigecycline, fluoroquinolones, aminoglycosides, colistin and rifampin [32, 42, 43]. Carbapenems have been regarded as the treatment of choice for severe *A. baumannii* infections [59, 60]. Strains respond briskly to carbapenems if they retain susceptibility to this antimicrobial class [15, 32]. However, increasing carbapenem-resistant *A. baumannii* strains are reported worldwide. Sulbactam is the most active of the *β*-lactamase inhibitors and has intrinsic antimicrobial activity against *A. baumannii* [41]. Sulbactam has shown promising results against *A. baumannii* strains with various susceptibility profiles [61–64], but its antimicrobial activity has declined substantially recently [65, 66]. Another option to treat *A. baumannii* infections is tigecycline. Tigecycline is a broad-spectrum antibiotic with bacteriostatic activity against *A. baumannii* [67, 68] and is commonly reserved for salvage therapy [69]. If *β*-lactams cannot be used, fluoroquinolones and aminoglycosides can be considered as potential treatment options [32]. For XDR *A. baumannii*, polymyxins are often the last treatment option. Unfortunately, polymyxins suffer from high rates of nephrotoxicity and neurotoxicity and possess no therapeutic window [32]. Recent in vitro models suggest that rifampicin maintains a high level of activity against *A. baumannii*, even in carbapenem-resistant strains [42, 70]. However, rifampicin should not be used in monotherapy as it induces the resistance of *A. baumannii* to itself [42].

In the present case, although meropenem and cefoperazone/sulbactam, which were among treatment options to *A. baumannii* infections [32, 42, 43], were used in this case. However, these antibiotics were not used according to the treatment guidelines for *A. baumannii* and may not reach the minimum inhibitory concentration (MIC). In addition, the *A. baumannii* strains in this case report may be carbapenem-resistant, MDR, or even XDR, often leading to very high mortality.

## Conclusions

This report presents a case of *A. baumannii* identified by molecular DNA sequencing, which was initially misidentified as a FLA species. Medical workers should be aware that patients associated with mechanical ventilation, surgery and invasive procedures, and prolonged broad-spectrum antimicrobials, especially those who suffer from burns, have trauma, or are in the ICUs, are among high risk-population of *A. baumannii* infection. If these patients develop FUO, *A. baumannii* infection should be taken into consideration. Clinically, due to the use of empirical antibiotics, blood culture results may be negative, and pathogen morphology under microscopic examination is sometimes atypical. In that case, the causative pathogen should be identified by molecular DNA sequencing if possible.

## Additional files

Additional file 1: Multilingual abstracts in the five official working languages of the United Nations. (PDF 724 kb)

Additional file 2: **Figure 1.** Line chart showing a Eosinophil count, b Eosinophil percentage, c lymphocyte count, d Lymphocyte percentage, e Monocyte count, and f Monocyte percentage during hospitalization. The time periods when antibiotics and antiamebics were used are shown. (TIFF 2677 kb)

Additional file 3: **Figure 2.** Line chart indicating a Red Blood cell count, b Hemoglobin, c Hematocrit and d Blood platelet count during hospitalization. The time points of blood transfusion are shown (red arrow heads). The time periods when antibiotics and antiamebics were used are shown. (TIFF 1622 kb)

Additional file 5: **Figure 3.** PCR amplification using prokaryotic universal primers and eukaryotic universal primers. M: Size markers; 1: No sequencing data were obtained using primers pair 18S-1080F/18S-1578R; 2: Sequencing data obtained using primers pair 18S-F /18S-R; 3: Sequencing data obtained using primers pair 16 s-27F/16 s-1390R. (TIFF 1033 kb)

Additional file 6: **Table 1.** Comparison of the obtained sequence using prokaryotic universal primers pair 16 s-27F /16 s-1390R with the top ten Species from the Blast. (XLSX 9 kb)

Additional file 7: **Table 2.** Comparison of obtained sequence (using prokaryotic universal primers pair 16 s-27F /16 s-1390R) with Acinetobacter baumannii strain KAB05, complete genome. Query = obtained sequence; Sbjct = Acinetobacter baumannii strain KAB05, complete genome. (XLSX 12 kb)

## Abbreviations

AT: Antiamebic treatment
COSMZ: Compounded sulfamethoxazole and sulfadiazine
CSF: Cerebrospinal fluid
FLA: Free-living amoeba
FUO: Fever of unknown origin
ICU: Intensive care unit
MDR: Multidrug-resistant
MIC: Minimum inhibitory concentration
PCR: Polymerase chain reaction
PDR: Pandrug-resistant
TF: Treatment failure
XDR: Extensively drug-resistant
