# Supplementary material for: Postoperative infection caused by Acinetobacter baumannii misdiagnosed as a free-living amoeba species in a humeral head hemiarthroplasty patient: a case report
Source: Infect Dis Poverty. 2018 Mar 31;7:33. doi: 10.1186/s40249-018-0408-5 (PMC5890356; doi:10.1186/s40249-018-0408-5)
Supplement: Supplementary file 2 — Multilingual abstracts in the five official working languages of the United Nations. (PDF 724 kb) [file 40249_2018_408_MOESM1_ESM.pdf]

Translation of the abstract into the six official working languages of the United Nations

عدوى ما بعد الجراحة الناجمة عن بكتيريا أسينتوباكتر بوماني وتم تشخيصه بصورة خاطئة كأنه أحد أنواع الأميبيات حُرة المعيشة لمريض استبدال رأس عضد الورك الجزئي: تقرير حالة

جياكسين تانغ، هويمين تشو، لي تساى، تينغ تينغ تانغ، جيان تانغ، يوي هوا صن، مينغ ليو، كيرونغ داي، زيغوانغ تشياو وتشاو يو

الملخص

المعلومات الأساسية: بكتيريا أسينتوباكتر بومانيهي بكتيريا واسعة الانتشار مخيرة تتكون داخل الخلايا وهي بكتريا انتهازية. وقدراتها الفريدة من نوعها تسمح لها بالبقاء على قيد الحياة في مجموعة متنوعة من البيئات بما في ذلك مرافق الرعاية الصحية، مما يؤدي إلى العدوى في المستشفيات. كما أن قدرتها الاستثنائية على تطوير مقاومة ضد العديد من المضادات الحيوية لا يدع سوى القليل من الأدوية متاحة للعلاج. قد تم الإقرار بأنها السبب الرئيسي للالتهاب الرئوي في المستشفيات وتجرثم الدم في جميع أنحاء العالم.

عرض الحالة: في هذه الحالة، تم عرض امرأة تبلغ من العمر 73 عامًا تعاني من كسر عضدي داني من مجموعة نير 6. وبعد ست ساعات من إجراء جراحة استبدال رأس عضد الورك الجزئي أصيبت بحمى مستمرة. وكشف الفحص السريري أن الوظائف الحيوية كانت طبيعية. وكشفت الفحوصات المخبرية والفحوصات الإشعاعية فقط عن مستويات مرتفعة من البروكالسيتونين. ولم تكشف عينة زراعة دم عن نمو البكتيريا أو الفطريات. ولم تظهر المعالجة بالتبريد والعلاج بالمضادات الحيوية واسعة الطيف التجريبية أي تأثير واضح.

الاستنتاجات: الإبلاغ عن وجود عدوى ما بعد الجراحة الناجمة عن بكتيريا أسينتوباكتر بوماني. تم التعرف على مسبب العدوى عن طريق تسلسل الحمض النووي الجزيئي وفي البداية وقع خطأ في تحديده على أنه أحد أنواع الأميبيات حُرة المعيشة عبر الفحص المجهري. وتمت علاج المريضة بصورة خاطئة عبر أعطائها علاج مركب من مُضادات الأَميبا. استمرت تلك الأعراض لديها لأكثر من 4 أشهر وتبع ذلك وفاتها.

Translated from English version into Arabic by Um Sarah, proofread by Shaima Riad, through

TRANSLATORS
WITHOUT BORDERS

肱骨头置换术后鲍曼不动杆菌感染误诊为自由生活阿米巴感染一例

唐佳昕，朱淮民，蔡黎，汤亭亭，唐坚，孙月华，刘铭，戴尅戎，乔之光，俞超

摘要

引言：鲍曼不动杆菌是一种机会致病菌，它普遍存在，具有兼性胞内寄生特性。它独特的适应能力使其可以在包括医疗设施在内的多种环境中存活，常常引起医院感染。而它特有的对多重耐药性又使得仅有少数几种抗生素对其有效。在世界范围内，鲍曼不动杆菌感染已被视为引起医源性肺炎、菌血症的主要病原体。

病例介绍：本案中的患者是一位 73 岁女性患者，Neer 分型 VI 型肱骨近端骨折。在成功行肱骨头置换术后 6 小时后，出现稽留热。患者生命体征平稳，实验室检查及影像学检查仅发现降钙素原水平升高。血培养未发现细菌及真菌生长。然而，降温措施和经验性广谱抗生素治疗未能获得显著疗效。

结论：本文报道了一例肱骨头置换术后的鲍曼不动杆菌感染。起初，因显微镜观察结果的误导，该病原体被误诊为一种自由生活阿米巴，后经 DNA 测序得知该病原体实为鲍曼不动杆菌。由于误诊，在院期间使用抗阿米巴联合用药治疗。患者的症状持续超过 4 个月，最终治疗无效死亡。

Translated from English version into Chinese by Jiaxin Tang

Infection postopératoire causée par *Acinetobacter baumannii*, imputée par erreur de façon à une espèce d'amibe libre, consécutive à une hémiarthroplastie de tête humérale : une étude de cas

Jiaxin Tang, Huaimin Zhu, Li Cai, Tingting Tang, Jian Tang, Yuehua Sun, Ming Liu, Kerong Dai, Zhiguang Qiao et Chao Yu

Résumé

Contexte: *Acinetobacter baumannii* est une bactérie ubiquiste, intracellulaire facultative et opportuniste. Ses capacités uniques lui permettent de survivre dans des environnements très divers, entre autres dans les établissements de santé où elle provoque des infections nosocomiales. En outre, sa capacité exceptionnelle de développer une résistance à de nombreux antibiotiques ne laisse que peu d'options pour le traitement médicamenteux. Cette espèce bactérienne est reconnue comme une cause majeure de pneumonies et bactériémies nosocomiales dans le monde.

Présentation du cas: Le cas présenté ici est celui d'une femme de 73 ans, présentant une fracture de l'extrémité supérieure de l'humérus du groupe VI selon la classification de Neer. Six heures après avoir subi une hémiarthroplastie, elle a développé une fièvre continue. L'examen clinique a montré que les signes vitaux étaient normaux. Les analyses de laboratoire et les radiographies n'ont montré qu'une élévation du taux de procalcitonine. L'hémoculture n'a pas montré de prolifération bactérienne ou fongique. Un traitement antipyrétique et une antibiothérapie empirique avec un antibiotique à large spectre n'ont produit aucun effet apparent.

Conclusions: Nous rapportons une infection postopératoire causée par *Acinetobacter baumannii*. L'agent pathogène à l'origine de l'infection a été identifié par séquençage ADN. Il avait été initialement mal identifié, à l'examen au microscope, comme une espèce d'amibe libre. La patiente a été traitée de façon erronée par une thérapie combinée antiamibienne. Ses symptômes ont persisté pendant plus de 4 mois, à l'issue desquels elle est décédée.

Translated from English version into French by Ode Laforge, proofread by Suzanne Assenat, through

TRANSLATORS WITHOUT BORDERS

Послеоперационная инфекция, вызванная *Acinetobacter baumannii* и ошибочно диагностированная как свободно живущий вид амебы в плечевой головке пациента гемиартропластики: описание клинического случая

Цзясинь Тан, Хуаймин Чжу, Ли Цай, Тинтин Тан, Цзянь Тан, Юэхуа Сунь, Мин Лю, Кэжун Дай, Чжигуан Цяо и Чао Юй

Аннотация

История вопроса: *Acinetobacter baumannii* — это повсеместная факультативная внутриклеточная условно-патогенная бактерия. Ее уникальные способности позволяют ей

выживать в различных средах, включая медицинские учреждения, что может послужить причиной внутрибольничной инфекции. Ее исключительная способность вырабатывать устойчивость ко многим антибиотикам предоставляет ограниченные возможности для лечения лекарственными средствами. Эта бактерия была признана ведущей причиной внутрибольничной пневмонии и бактериемии во всем мире.

Представление клинического случая: В этом клиническом случае представлена 73-летняя женщина с переломом проксимального отдела плечевой кости VI группы Нира. Спустя шесть часов после успешно проведенной гемиартропластики у нее появилась постоянная лихорадка. Клинический осмотр выявил, что показатели жизненно важных функций были в норме. Лабораторные и рентгенологические исследования обнаружили только повышенный уровень прокальцитонина. В посеве крови не было обнаружено роста бактерий или грибков. Холодильная обработка и эмпирическая терапия антибиотиками широкого спектра действия не показала видимого воздействия.

Выводы: Было обнаружено, что причиной послеоперационной инфекции является *Acinetobacter baumannii*. Возбудитель инфекционного заболевания был обнаружен с помощью молекулярного секвенирования ДНК и изначально был ошибочно продиагностирован путем гистологического исследования как свободно живущий вид амебы. Пациенту было ошибочно назначено лечение комбинированной терапией с применением противоамёбных препаратов. Ее симптомы сохранялись более 4 месяцев и в итоге привели к смерти.

Translated from English version into Russian by Ira Kulinevych, proofread by Liudmila Tomanek, through

Infección posoperatoria causada por *Acinetobacter baumannii* mal diagnosticada como una especie de ameba de vida independiente en la cabeza del húmero de una paciente con hemiartroplastia: estudio de caso

Jiaxin Tang, Huaimin Zhu, Li Cai, Tingting Tang, Jian Tang, Yuehua Sun, Ming Liu, Kerong Dai, Zhiguang Qiao and Chao Yu

Resumen

Antecedentes: *Acinetobacter baumannii* es una bacteria ubicua, intracelular facultativa y oportunista. Sus habilidades le permiten sobrevivir en una amplia gama de entornos, en establecimientos de atención médica inclusive, lo que provoca infecciones nosocomiales. Su capacidad excepcional de desarrollar resistencia a múltiples antibióticos deja pocos medicamentos que puedan tratarla. Ha sido reconocida la causa principal de neumonía nosocomial y bacteriemia en todo el mundo.

Presentación del caso: En esta ocasión, una mujer de 73 años presentó una fractura de húmero proximal Neer Group VI. Seis horas luego de una hemiartroplastia realizada con éxito, contrajo fiebre continua. Se reveló a través de un exámen clínico que los signos vitales eran normales. Los estudios radiográficos y de laboratorio solo dieron a conocer altos niveles de procalcitonina. El hemocultivo no

demostró crecimiento bacteriano o fúngico alguno. Los tratamientos con frío y antibióticos de amplio espectro con base empírica no surtieron efecto aparente.
Conclusiones: Diagnosticamos una infección posoperatoria provocada por *Acinetobacter baumannii*. Se distinguió el agente patógeno infeccioso mediante secuenciación del ADN molecular y se identificó al principio como una especie de ameba de vida independiente en exámenes microscópicos. Se le practicó de forma errónea tratamiento antiamebiano combinado a la paciente. Sus síntomas perduraron más de 4 meses y por último, produjeron su muerte.

Translated from English version into Spanish by Constanza DN, proofread by Susana Rosseli, through

TRANSLATORS
WITHOUT BORDERS
