# Supplementary figures and images for: Postoperative infection caused by Acinetobacter baumannii misdiagnosed as a free-living amoeba species in a humeral head hemiarthroplasty patient: a case report
Source: Infect Dis Poverty. 2018 Mar 31;7:33. doi: 10.1186/s40249-018-0408-5 (PMC5890356; doi:10.1186/s40249-018-0408-5)

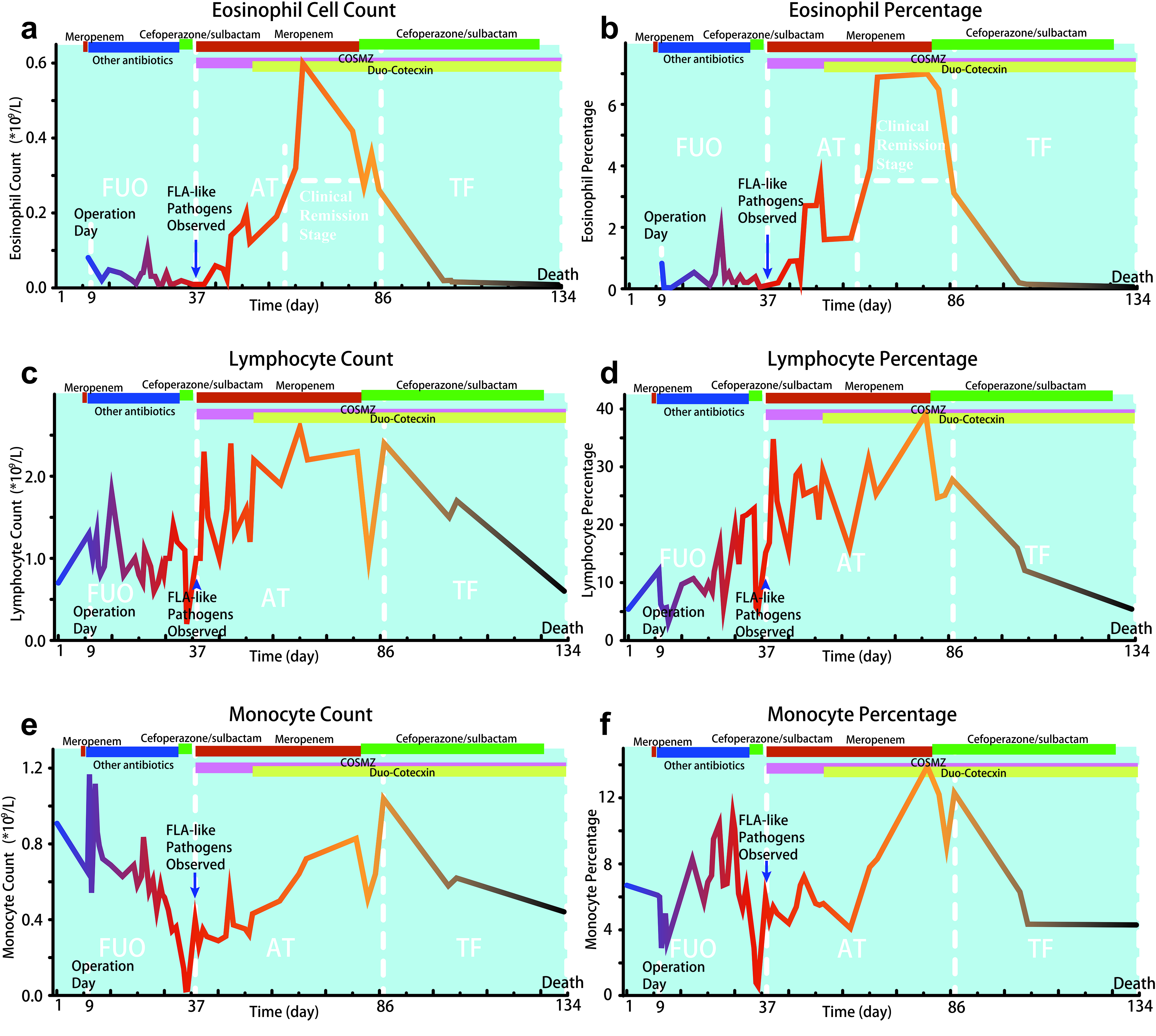

Supplement: Supplementary file 3 — Figure 1. Line chart showing a Eosinophil count, b Eosinophil percentage, c lymphocyte count, d Lymphocyte percentage, e Monocyte count, and f Monocyte percentage during hospitalization. The time periods when antibiotics and antiamebics were used are shown. (TIFF 2677 kb) [file 40249_2018_408_MOESM2_ESM.tif]

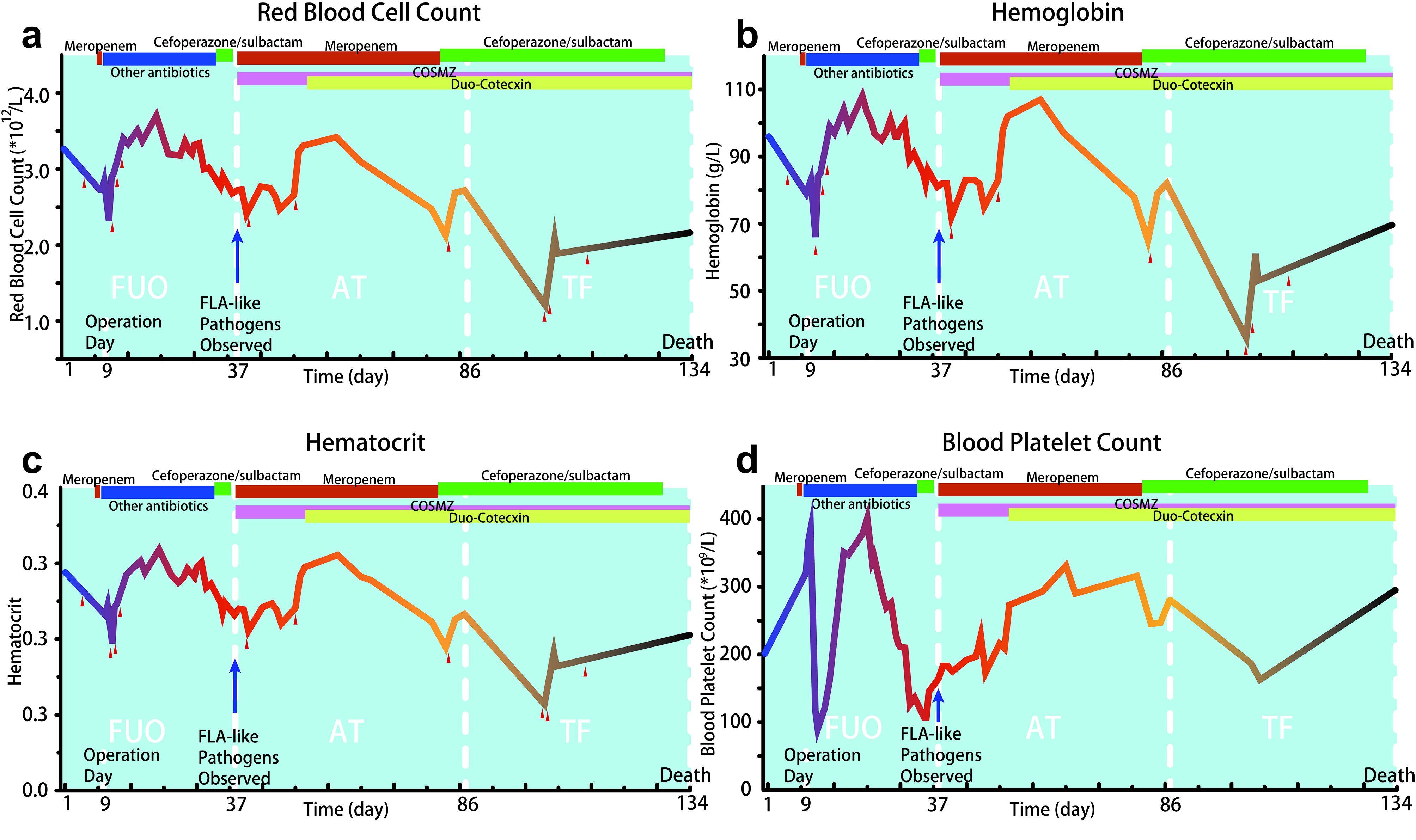

Supplement: Supplementary file 4 — Figure 2. Line chart indicating a Red Blood cell count, b Hemoglobin, c Hematocrit and d Blood platelet count during hospitalization. The time points of blood transfusion are shown (red arrow heads). The time periods when antibiotics and antiamebics were used are shown. (TIFF 1622 kb) [file 40249_2018_408_MOESM3_ESM.tif]

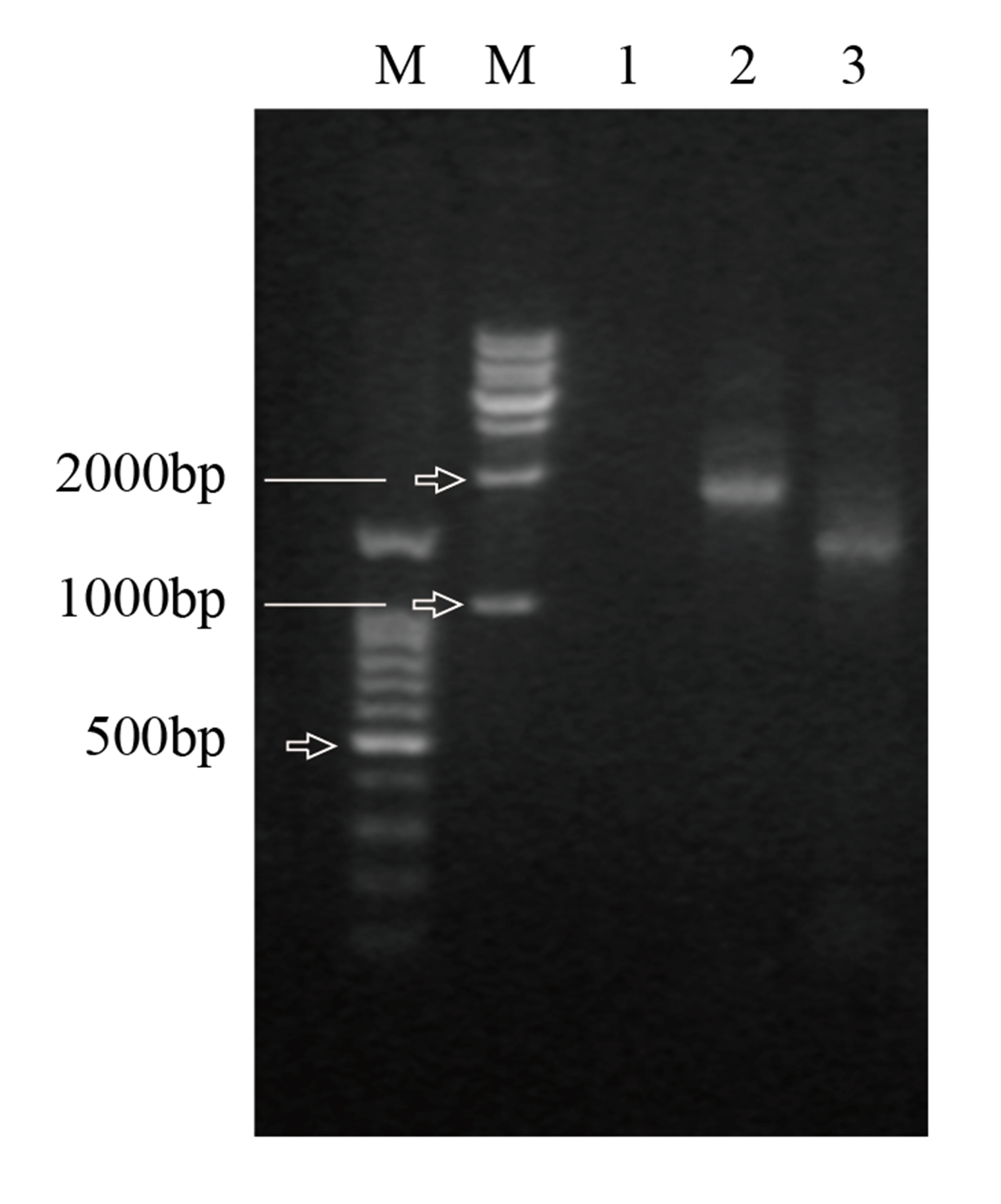

Supplement: Supplementary file 5 — Figure 3. PCR amplification using prokaryotic universal primers and eukaryotic universal primers. M: Size markers; 1: No sequencing data were obtained using primers pair 18S-1080F/18S-1578R; 2: Sequencing data obtained using primers pair 18S-F /18S-R; 3: Sequencing data obtained using primers pair 16 s-27F/16 s-1390R. (TIFF 1033 kb) [file 40249_2018_408_MOESM5_ESM.tif]
